# Digital health literacy and digital engagement for people with severe mental ill health across the course of the COVID-19 pandemic in England

**DOI:** 10.1186/s12911-023-02299-w

**Published:** 2023-09-26

**Authors:** P Spanakis, B Lorimer, E Newbronner, R Wadman, S Crosland, S Gilbody, G Johnston, L. Walker, E Peckham

**Affiliations:** 1 Department of Health Sciences, University of York, York, UK; 2 Department of Psychology, University of Crete, Rethymnon, Greece; 3 Independent Peer Researcher, Clackmannan, UK; 4 School of Health and Psychological Sciences, City, University of London, London, UK; 5 School of Medical and Health Sciences, Bangor University, Bangor, UK

**Keywords:** Severe mental illness, Schizophrenia, Bipolar disorder, Internet, Digital Health Literary, Digital divide

## Abstract

**Background:**

An unprecedented acceleration in digital mental health services happened during the COVID-19 pandemic. However, people with severe mental ill health (SMI) might be at risk of digital exclusion, partly because of a lack of digital skills, such as digital health literacy. The study seeks to examine how the use of the Internet has changed during the pandemic for people with SMI, and explore digital exclusion, symptomatic/health related barriers to internet engagement, and digital health literacy.

**Methods:**

Over the period from July 2020 to February 2022, n = 177 people with an SMI diagnosis (psychosis-spectrum disorder or bipolar affective disorder) in England completed three surveys providing sociodemographic information and answering questions regarding their health, use of the Internet, and digital health literacy.

**Results:**

42.5% of participants reported experiences of digital exclusion. Cochrane-Q analysis showed that there was significantly more use of the Internet at the last two assessments (80.8%, and 82.2%) compared to that at the beginning of the pandemic (65.8%; ps < 0.001). Although 34.2% of participants reported that their digital skills had improved during the pandemic, 54.4% still rated their Internet knowledge as being fair or worse than fair. Concentration difficulties (62.6%) and depression (56.1%) were among the most frequently reported symptomatic barriers to use the Internet. The sample was found to have generally moderate levels of digital health literacy (M = 26.0, SD = 9.6). Multiple regression analysis showed that higher literacy was associated with having outstanding/good self-reported knowledge of the Internet (ES = 6.00; 95% CI: 3.18–8.82; p < .001), a diagnosis of bipolar disorder (compared to psychosis spectrum disorder – ES = 5.14; 95% CI: 2.47–7.81; p < .001), and being female (ES = 3.18; 95% CI: 0.59–5.76; p = .016).

**Conclusions:**

These findings underline the need for training and support among people with SMI to increase digital skills, facilitate digital engagement, and reduce digital engagement, as well as offering non-digital engagement options to service users with SMI.

**Supplementary Information:**

The online version contains supplementary material available at 10.1186/s12911-023-02299-w.

## Introduction

Digital technologies are increasingly used for research and intervention purposes in people with severe mental ill health (SMl; schizophrenia spectrum and bipolar disorder). For example, smartphones have recently been used as a tool for real-time data collection related to psychiatric symptoms in real-world settings, also known as “digital phenotyping” [1–4]. A recent review identified 63 digital technologies developed for people with schizophrenia spectrum disorders that offered a range of services such as medication adherence, therapy, cognitive and social deficits training, and health behaviour change support [5]. The COVID-19 pandemic and its associated restrictions on social contact led to an unprecedented acceleration in the provision of digital mental health services [6]. For example, 80% of people with SMI in England recently reported that their mental health service provision changed from face-to-face to remote (over the phone or online) [7]. Additionally, it has been argued that a full return to traditional face-to-face services is unlikely [8, 9].

Despite these prospects, not all people with SMI engage with digital technologies, with some not using them at all and others using them in a restricted manner. During the COVID-19 pandemic, a period when many people were heavily relying on the internet to complete their daily activities, 39.5% of people with SMI in the UK were not using the internet, as opposed to 5% in the general population, highlighting the existence of a digital divide [10, 11]. Digital exclusion may adversely affect people with SMI. For example, this population faces shorter life expectancy compared to the non-SMI population due to long-term illnesses [12, 13], and people with SMI often report feeling lonely [14]. These inequalities may be further exacerbated by low level of engagement with health services and socialization resources online [15].

Digital exclusion is a complex phenomenon involving multiple factors such as lack of internet access, lack of digital skills and financial barriers to paying for the Internet (data poverty; [16]). Previously, although most people with SMI reported having access to the Internet and sufficient data to cover their needs, about 42.2% lacked foundation digital skills [17]. Indeed, lack of skills/difficulty in using the Internet is commonly reported as a barrier to accessing the Internet [10, 18, 19]. One particularly important digital skill is digital health literacy which refers to a person’s ability to find and understand health-related information online and apply this knowledge to make healthcare decisions and self-manage their conditions [20]. Data on levels of digital health literacy among people with SMI is limited, especially for the United Kingdom population. Findings from some international studies suggest low to moderate levels among people with schizophrenia [20], but higher levels for people with bipolar disorder [21].

Good health literacy in general, regardless of digital means, provides a better understanding of medical information and treatment-related materials. As such, low levels of health literacy among people with SMI have been associated with low service utilization and treatment adherence, as well as poor self-management and worse health outcomes [22]. It is also important to note that people with low health literacy often struggle to manage chronic illnesses [23]. This is of particular importance for people with SMI who often suffer not only from their long-term mental illnesses but also from comorbid long-term physical illnesses [12]. In a time of increased digitalization of health services, a lack of digital health literacy may contribute to the aforementioned health inequalities in people with SMI.

This study aimed to explore how the use of the Internet has changed from the start of the pandemic until the present time in people with SMI. The study also sought to examine the current level of various related experiences and skills, such as digital exclusion, symptomatic barriers to internet engagement, and digital health literacy. An additional aim was to understand the sociodemographic and health correlates of digital health literacy.

## Methods

### Design and participants

The Optimising Wellbeing in Self-Isolation (OWLS) study was set up in 2020 to longitudinally explore the effects of the COVID-19 pandemic and associated restrictions on people with SMI. Thus far, the OWLS study has primarily included the completion of three surveys since the beginning of the pandemic, with each survey being completed by participants by telephone, online, or by hard copy (depending on participant preference). The design and data analysis for the present study was pre-registered on Open Science Framework (10.17605/OSF.IO/KNV7H). Ethical approval for the OWLS study was granted by the Health Research Authority North-west – Liverpool Central Research Ethics Committee (REC reference 20/NW/0276) and Wales Research Ethics Committee 4 (REC reference 21/WA/0239).

**Fig. 1:**
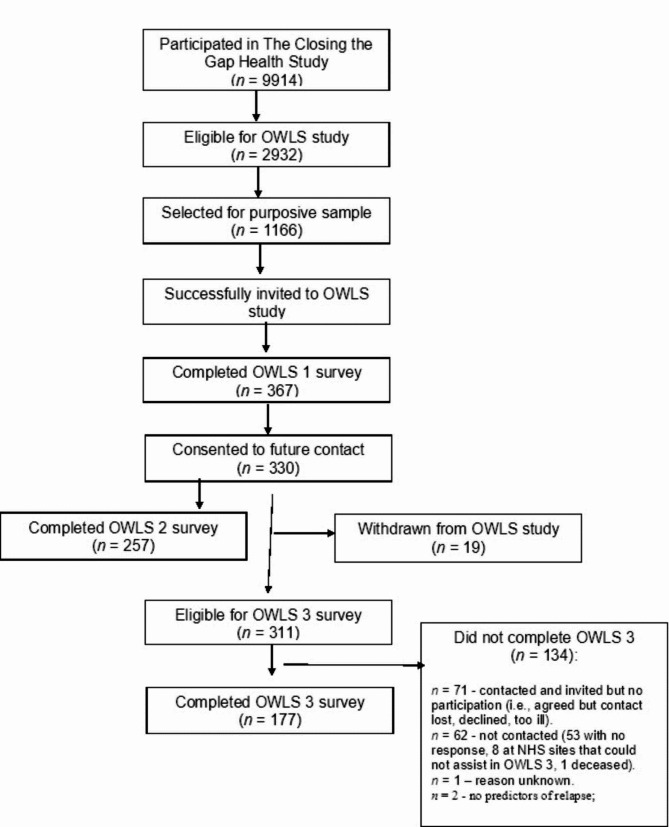
Recruitment Process for OWLS 3 Study

The full methods regarding study recruitment have been reported elsewhere [17]. To summarise, a subsample of people who had taken part in The Closing the Gap Health Study (CtG; 2016–2020) were invited to take part in the OWLS study. The CtG comprised *N* = 9914 people aged 18 and over who had a documented diagnosis of schizophrenia or delusional/psychotic illness (ICD 10 F20.X -F29.X or DSM equivalent) or bipolar disorder (ICD F31.X or F21.X or DSM equivalent). To be eligible to take part in the OWLS study, participants in CtG must have provided contact details and consented to be contacted again for further research, along with being originally recruited from a site that had the capacity to collaborate on the OWLS study. A total of *n* = 2932 participants were identified as eligible and a purposive sample of *n* = 1166 was then selected to be contacted and invited to participate in the OWLS study (Fig. 1). This purposive sample was selected based on the time of recruitment to the CtG study (e.g., recent recruitment to ensure valid contact details and familiarity with the research team), as well as gender, age, ethnicity, and primary vs. secondary care (to reflect the diversity of the population).

A total of *n* = 688 participants (59.0%) were successfully contacted and invited to take part in the OWLS study. We were unable to contact the other *n* = 478 (41%) due to missing or invalid contact details or due to the participants not responding to calls or emails. Those who were interested in taking part were then offered the option of completing an initial survey (‘OWLS 1’), and those who participated were asked if they were willing to take part in follow-up surveys. A total of *n* = 367 participants completed the OWLS 1 survey between July and December 2020, with *n* = 330 consenting to be contacted again in the future. The second OWLS study survey (‘OWLS 2’) was conducted between January and March 2021, with *n* = 257 participants completing this survey. During OWLS 2, *n* = 19 participants withdrew their consent to be contacted again for further surveys, consequently leaving a total of *n* = 311 participants who could be invited to participate in the third wave of the OWLS study (‘OWLS 3’) that took place between October 2021 and February 2022. The participants who completed the OWLS 3 survey during this period represent the primary sample for the results presented in this article.

### Measures

The OWLS 3 questionnaire (see Additional File 1) was developed in conjunction with a lived experience panel who both provided suggestions on items to include in the survey and piloted the questionnaire.

### Sociodemographic variables

Information related to each participant’s age, gender (male, female, or transgender), and ethnicity was obtained during CtG. Due to the limited number of participants with an ethnicity other than White, a binary variable for ethnicity was computed for the purposes of statistical analyses in this study (White or other than White). Moreover, participants’ level of neighbourhood deprivation was determined by linking their home postcode to the English Index of Multiple Deprivation (IMD; [24], which is a measure of relative deprivation for small geographic areas. The IMD ranks each area from most to least deprived, and indices are aggregated in this study into quintile groups (where 1 = most deprived, 5 = least deprived). Participants’ postcodes were updated using information collected over the course of the three OWLS surveys.

### Health variables

During CtG, some participants provided consent for their health records to be inspected. These records were reviewed to obtain each participant’s SMI diagnosis, which was subsequently categorized into psychosis spectrum disorders (including schizophrenia, schizoaffective, or any other psychotic disorder), bipolar disorder, or other SMI. This latter category included participants who were eligible for CtG based on a psychosis or bipolar disorder diagnosis, which was later changed in their health records to something different (e.g., depressive disorder with psychotic features). Where a participant had not provided consent to access their medical records or insufficient identifiable information had been supplied, the diagnosis was coded as “not recorded” and deemed missing.

Meanwhile, in the OWLS 3 survey, participants were asked to record whether they had any physical health conditions, and a binary variable was computed related to physical comorbidity (any comorbidity, or no comorbidity). In addition, participants were asked questions related to their engagement in three separate health risk behaviours (smoking, physical inactivity, and limited consumption of fruit and vegetables). For each behaviour, participants were deemed to be engaging in a specific risk behaviour if they reported that they smoke tobacco, do not meet physical activity guidelines (i.e., being active less than every other day), or do not meet guidelines for the consumption of fruit and vegetables (i.e., eating less than five portions of fruit or vegetables per day – [25]). An index encompassing all three health risk behaviours was computed, with this being the total number of health risk behaviours reported by participants.

### Digital variables

**Daily Internet Use (OWLS 1, 2, 3).** In each of the three OWLS surveys participants were asked if they used the Internet to do some of their daily activities (e.g., finding information, buying groceries, paying bills, etc.). In OWLS 2 and OWLS 3, this question was asked in relation to the previous 12 months, while in OWLS 1, this question was asked in relation to the time since the commencement of the pandemic. Participants could respond “yes, a lot”, “yes, a little”, or “no”, and a binary variable was computed for this study (yes or no). During OWLS 3, *n* = 20 participants were mistakenly presented with a differently worded question, which asked whether they had used the Internet for anything over the previous year. These participants’ responses to other questions in the survey were examined, and this enabled a response to the original question to be determined for *n* = 9 participants. Information regarding daily Internet use could be not determined from other responses for the remaining *n* = 11 participants, and these participants were therefore classed as ‘missing’ for this variable.

**Internet Knowledge and Digital Skills Improvement (OWLS 3).** Participants were asked to rate their knowledge of the internet with the following response options: “outstanding”; “good”; “fair”; “poor”; “bad”; or “don’t know/can’t say”. A binary variable for Internet knowledge was computed for some analyses conducted (outstanding/good or fair/poor/bad), with those who responded “don’t know/can’t say” deemed to have missing information. Participants were also asked if they believed that their digital skills had improved because of the pandemic, with the following options being available: “yes”; “no, although I do feel that they need improving”; “no, but I do not feel they need improving”; or “don’t know/prefer not to say”.

**Experience of Digital Exclusion (OWLS 3).** An open-ended question asked participants if they believed that a lack of digital skills or access prevented them from being able to do something that they needed or wanted to do. A simple qualitative inductive content analysis was conducted to identify the activities or tasks of most concern. The analysis was conducted manually as the amount of data generated by the free text question was modest and so did not require the use of qualitative data analysis software.

**Symptomatic Barriers to Internet Use (OWLS 3).** All participants were provided a list of common symptoms associated with SMI and were asked to record the extent to which each has obstructed their ability to use the Internet. Possible responses included: “almost never”, “a few times”, and “many times”. All symptoms asked can be found in Fig. 3.

**Digital Health Literacy (OWLS 3).** Participants’ levels of digital health literacy were assessed by the eight-item eHealth Literacy Scale (eHEALS; Norman and Skinner [26], adapted by Choi and DiNitto [27]). Each item is scored on a 1–5 Likert scale, and the total score across all items is calculated for each participant. Higher total scores indicate higher levels of Digital Health Literacy (range = 8–40). The eHEALS has been previously used to assess digital health literacy in people with SMI and the validity of the tool has been demonstrated across different samples [20, 28].

### Analysis

The analysis for the whole OWLS 3 study was preregistered in Open Science Framework (10.17605/OSF.IO/KNV7H). The research questions related to the working package reported in this paper are described in Sect. 2.3, subsections 2.3.1 to 2.3.6. All analyses were performed using R Statistical Software (R Core Team 2022, version 4.1.2). Descriptive statistics were used to profile demographic, health, and digital skills information. To investigate potential selection biases, participants who completed the OWLS 3 survey were compared to participants who were eligible to be invited to take part but did not participate for whatever reason in terms of age, gender, ethnicity, IMD, and diagnosis. Pearson’s chi-square tests were used to compare categorical data, while Welch’s t-tests or Mann-Whitney U tests were used to compare continuous variables, depending on distributions.

In addition, a Cochran’s Q test was conducted to investigate whether the proportion of how many participants reported using the Internet for daily activities changed over the course of the pandemic (i.e., over the completion of OWLS 1, OWLS 2, and OWLS 3 surveys). Post-hoc analyses were conducted using pairwise McNemar tests to identify any specific significant differences. Finally, a multiple linear regression was conducted to investigate potential factors associated with Digital Health Literacy. Eight variables were included in the model: age, gender, ethnicity, IMD, diagnosis, physical health comorbidity, cumulative index of health, and self-reported Internet knowledge. The variables of age, IMD, and a cumulative index of health were treated as continuous in nature, while the remaining were treated as categorical variables. Before applying Cochran’s Q test and the regression model, nonparametric missing value imputation was conducted using the R package *missForest* [29]. MissForest is an algorithm based on the machine learning approach of Random Forest, which can impute missing values in mixed-type datasets (i.e., contains both continuous and categorical variables), and has been demonstrated to be effective at handling missing values in variables that have up to 30% missing information [30]. As sensitivity analyses, Cochran’s Q test and regression model were conducted again using only those participants with complete information.

### Change from pre-registration

It was initially intended to also investigate alcohol consumption as an additional health risk behaviour, with this being included in the calculation of the cumulative index of health behaviours. However, many participants (~ 34%) did not complete the AUDIT-C (i.e., a three-item measure of at-risk drinking; [31]). Considering that other variables related to health risk behaviours had substantially fewer missing values (*n* = 0–1), it was deemed that missing values for the AUDIT-C were not missing at random. Therefore, alcohol consumption was not examined further, as it was deemed inappropriate with the available data. Consequently, the cumulative index of health behaviours was calculated using information related to physical activity, consumption of fruit and vegetables, and smoking only.

## Results

Between October 2021 and February 2022, *n* = 248 participants were successfully contacted and invited to participate in OWLS 3, with *n* = 177 (71.4%) completing the survey. The flow of participants through the OWLS study is illustrated in Fig. 1.

Table 1 describes the sociodemographic and health information of the participants. Demographic and health information statistics (i.e., means, standard deviations, and percentages) were calculated using only those cases with full, relevant information. The mean age was 52.2 (*SD* = 15.1; range = 23–85; IQR = 42–64), with 50.3% of the sample being male and 87.0% being of white ethnicity. Most participants lived in neighbourhoods that had moderate levels of deprivation (*M* = 2.9; *SD* = 1.4; range = 1–5; IQR = 2–4). In terms of health, 52.8% of participants had a diagnosis of psychosis spectrum disorder, and 68.4% reported having physical health comorbidity. Participants also reported that they engaged in a mean of 0.9 health risk behaviours (*SD* = 0.8, range = 0–3, IQR = 0–1), with not meeting guidelines for the consumption of fruit and vegetables being the most frequently engaged behaviour (74.6%).

**Table 1:** Socio-Demographic and Health Information of the Sample (N = 177)

Variable	N (%^a^) / M (SD)	Missing N
**Socio-Demographic Information**		
Gender, *n* (%)		0
Male	89 (50.3)	
Female	85 (48.0)	
Transgender	3 (1.7)	
Age, *M* (*SD*)	52.2 (15.1)	0
Ethnicity, *n* (%)		0
White	154 (87.0)	
Mixed	8 (4.5)	
South Asian	4 (2.3)	
African	2 (1.1)	
Caribbean	2 (1.1)	
Other	7 (4.0)	
Index of Multiple Deprivation, *M* (*SD*)	2.9 (1.4)	4
Index of Multiple Deprivation Quintiles, *n* (%)		4
Very Highly Deprived (IMDQ = 1)	37 (21.4)	
Highly Deprived (IMDQ = 2)	41 (23.7)	
Moderately Deprived (IMDQ = 3)	35 (20.2)	
Lowly Deprived (IMDQ = 4)	28 (16.2)	
Very Lowly Deprived (IMDQ = 5)	32 (18.5)	
**Health Information**		
Diagnosis, *n* (%)		16
Psychosis Spectrum Disorder	85 (52.8)	
Bipolar Disorder	62 (38.5)	
Other SMI	14 (8.7)	
Physical Health Comorbidity, *n* (%)		0
At Least One	121 (68.4)	
None	56 (31.6)	
Cumulative Index of Health Behaviours, *M* (*SD*)	0.9 (0.8)	0
Eating < 5 Fruit or Vegetables Per Day, *n* (%)	132 (74.6)	0
Exercising Less Than Every Other Day, *n* (%)	89 (50.6)	1
Smoking Tobacco, *n* (%)	36 (20.3)	0

^a^ Percentages calculated using only those cases with full data (i.e., excluding missing)

### Potential selection biases

Comparisons of the characteristics of participants who did (*n* = 177) and who did not (*n* = 134) complete the OWLS 3 survey indicated that White participants (χ^2^ = 4.76, df = 1, p = .029) were more likely to complete the survey than other than White. Specifically, 87.0% of participants who completed the survey were White, while 77.6% of those who did not complete the survey were White. There were no significant differences (p > .05) in terms of age, gender, diagnosis, and IMD.

### Internet use

**Fig. 2:**
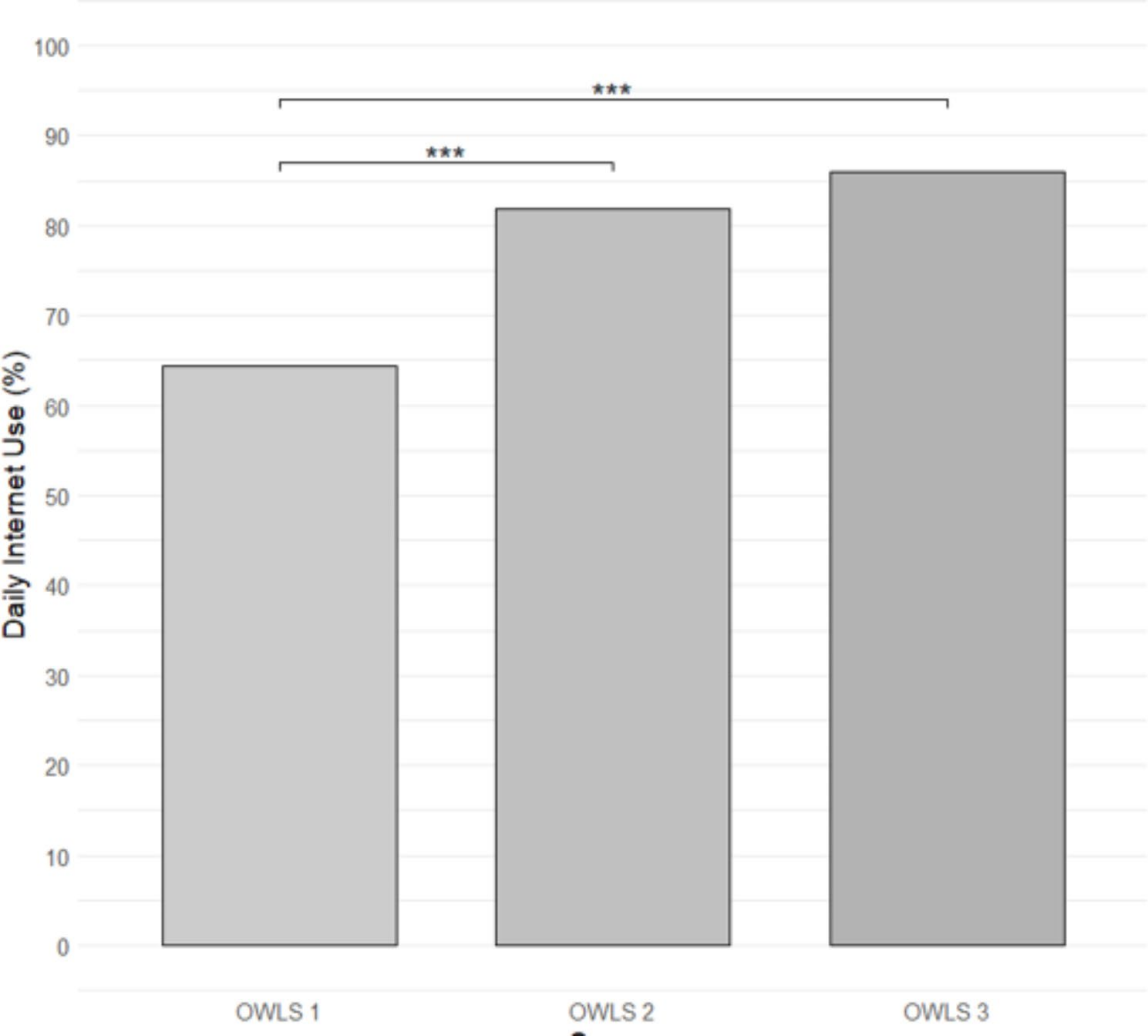
Proportions of participants in each OWLS survey who report using the Internet to do some of their daily activities at least some of the time (n = 177; imputed data). *** indicates statistically significant difference between two proportions (p < .001). Note – See Supplementary Materials B for results when analyses repeated with only those n=146 participants with complete information

A total of *n* = 141 participants (84.9% after excluding *n* = 11 with missing data) reported using the Internet to do some of their daily activities at least some of the time. Of the *n* = 146 participants who responded to the relevant question in all three OWLS surveys, 65.8%, 80.8%, and 84.9% reported using the Internet daily in OWLS 1, OWLS 2, and OWLS 3, respectively. When utilizing imputed data for all participants (*n* = 177), a Cochran’s Q test identified a significant difference between the three-time points in terms of proportions of Internet use (*Q* = 50.1, *df* = 2, *p* < .001). Specifically, posthoc pairwise McNemar tests identified that participants reported using the Internet significantly less (both *p* < .001) during OWLS 1 than during OWLS 2 or OWLS 3 (see Fig. 2). No difference was found between OWLS 2 and OWLS 3 (*p* = .108). Similar findings were observed when repeating the Cochran’s Q test with only those *n* = 146 participants with complete information (see Additional File 2).

Table 2 reports participants’ responses related to their self-reported knowledge of the Internet, whether they believed that their skills had improved over the course of the pandemic, and whether they ever experienced digital exclusion. As shown in the table, most participants (54.4%) reported their Internet knowledge as being ‘fair’ or worse. Additionally, 34.2% reported that their digital skills had improved due to the pandemic, while 31.6% indicated that their skills did not improve, and that improvement was required.

**Table 2:** Participants’ Responses to Questions Related to Digital Skills (N = 177)

	N (%^a^)	Missing N
Self-reported rating of Internet knowledge		8^b^
Outstanding	25 (14.8)	
Good	52 (30.8)	
Fair	53 (31.4)	
Poor	19 (11.2)	
Bad	20 (11.8)	
Digital skill improvement due to pandemic		22^c^
Yes	53 (34.2)	
No, but improvement not needed	53 (34.2)	
No, and improvement needed	49 (31.6)	
Functional impairment due to limited digital skills		3
Yes	74 (42.5)	
No	100 (57.5)	

^a^ Percentages calculated using only those cases with full data (i.e., excluding missing)

^b^ Total includes participants who responded “Don’t Know/Can’t Say”

^c^ Total includes participants who responded “Don’t Know/Prefer Not to Say”

### Digital exclusion

A total of 42.5% of participants (*n* = 74) reported that a lack of digital skills had prevented them from doing something necessary at least once. Reviewing the free-text responses from the *n* = 56 participants who provided information on what they had been unable to do, the following patterns emerged.

Of those who responded, n = 49 described specific activities or tasks that they had been unable to do, and six broad areas were highlighted: Life Admin; Financial Tasks; Shopping; Social and Learning; Leisure; and Information Seeking. The area most often highlighted (by 21.5% of respondents) was Life Admin. This included a range of tasks and activities that are essential to everyday life, such as dealing with official bodies (e.g., housing providers, local government, etc.), ordering repeat prescriptions, making appointments and bookings, and changing/communicating with utility providers. Example responses are: *“Trying but failing to register for online repeat prescriptions”*, and *“Council Tax went digital and energy company required* [me] *to submit meter readings – both difficult”*.

Dealing with financial matters and shopping online were also areas of concern. For example, 14.2% of respondents said that they struggled with financial tasks, such as internet banking, paying bills online, sorting out benefits, and making phone top-ups. Two brief quotations illustrate this: *“Can’t access my Tax Account with HMRC, even though I know their calculations are wrong!”*; “[unable to] *check benefits online”*. Similarly, 12.5% found it difficult to shop online. This included buying goods direct from online shops and suppliers, but also using auction sites such as eBay to buy or bid for items, and the use of different payment methods, for example: *“Trying to order presents when the option via PayPal is not available”*.

Other respondents (10.7%) reported having problems using the internet for social and learning activities, such as joining social/spiritual meetings by Zoom, taking part in online courses, or engaging in social media. For example, one respondent simple reported *“Involvement in courses”*, whilst another reported *“Trying to navigate Facebook”*. The same proportion (10.7%) said that they struggled with leisure activities, such as accessing streaming services (e.g., Netflix, Spotify), or downloading photographs. Two short quotations illustrate this: *“Photographic things – end up going round in circles”* and *“Sports available online and I couldn’t access it at all”*. Lastly, a few respondents (5.3%) noted that they had difficulty finding information on the internet.

### Symptomatic barriers to internet use

**Fig. 3:**
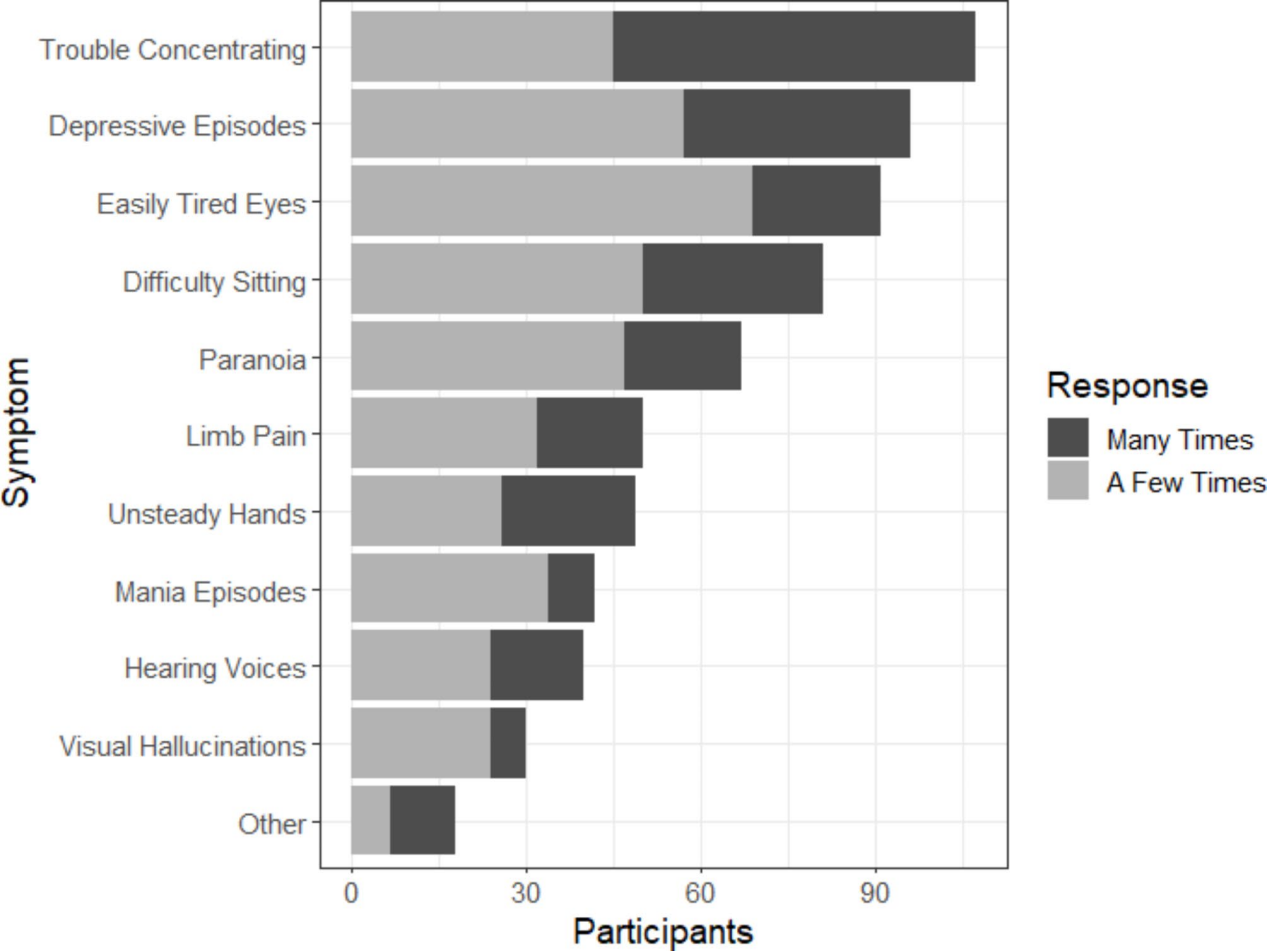
Proportions of participants who self-report that specific symptoms act as barriers to Internet use (n=171). Note - ‘Other’ symptoms included: fatigue, low motivation, anxiety, migraines, post-stroke symptoms

Figure 3 summarises the specific symptoms that participants highlighted as being frequent barriers to Internet use. A total of *n* = 166 participants provided complete responses to this question, with *n* = 5 providing responses to at least half the symptoms (missing responses for these participants were deemed as “Almost Never” responses). The most reported symptomatic barriers to Internet use were trouble concentrating (62.6% reports of this symptom limiting Internet use at least a few times), experiencing depressive episodes (56.1%), and easily tired eyes (53.2%). These were the only symptoms reported to limit Internet use at least a few times by a majority of the sample.

### Digital health literacy

Seven participants did not respond to all items within the eHEALS questionnaire. For the *n* = 170 participants who did, an average score of 26.0 (*SD* = 9.6, range = 8–40, IQR = 19–32) was calculated. Before conducting the multiple linear regression to investigate factors associated with Digital Health Literacy, *n* = 3 transgender participants were excluded, due to there being too few participants of this gender to enable appropriate analysis. Consequently, imputed data from a total of *n* = 174 participants were available, and Table 3 summarises the results from the conducted linear regression. It was found that having outstanding or good self-reported knowledge of the Internet, having a diagnosis of bipolar disorder (compared to psychosis spectrum disorder), and being female were significantly associated with having higher levels of Digital Health Literacy. Similar findings were observed when repeating the regression model using only those n = 142 participants with complete information (see Additional File 1), however, being female was no longer found to be significantly associated with Digital Health Literacy while age was found to be significantly associated.

**Table 3:** Factors associated with Digital Health Literacy (n = 174)

	Estimate	SE	95% CI		p
			LL	UL	
Intercept	22.29	3.04	16.30	28.29	< 0.001*
Age	-0.09	0.05	-0.19	< 0.01	0.057^a^
Gender - Female	3.18	1.31	0.59	5.76	0.016*^b^
Ethnicity – Other than White	0.24	1.91	-3.52	4.00	0.899
Index of Multiple Deprivation	0.47	0.47	-0.46	1.39	0.321
Diagnosis - Bipolar	5.14	1.35	2.47	7.81	< 0.001*
Diagnosis – Other SMI	1.51	2.45	-3.33	6.35	0.538
Physical Health Problem – Having One	-0.55	1.39	-3.30	2.20	0.694
Cumulative Index of Health	1.15	0.83	-0.49	2.80	0.168
Internet Knowledge – Outstanding/Good	6.00	1.43	3.18	8.82	< 0.001*

a. Age statistically significant in sensitivity analysis (regression model with complete case data only; see Supplementary Table S1)

b. Gender not statistically significant in sensitivity analysis (see Supplementary Table S1)

* Statistically significant when tested against an alpha value of 0.05

## Discussion

This study examined Internet engagement among people with SMI across the different stages of the pandemic, from its outset in 2020 until the summer of 2022. Participants were asked about changes in their use of the Internet, current levels of knowledge about the Internet, experiences of digital exclusion, and digital health literacy. Sociodemographic and health-related correlates of digital health literacy were also identified.

Experiences of digital exclusion were reported by 42.5% of our participants (i.e., not being able to do things that they wanted/needed to do due to a lack of digital skills or access). We have previously found that lack of digital skills seems to affect our participants at a greater level than lack of access (42.2% lacked foundation skills while 85.9% and 83.5% had access to digital devices and internet connection respectively – Spanakis et al., [17]). However, we do not know how much each of these factors contributed to the experience of digital exclusion and future studies should focus on understanding this further.

The number of people with SMI reporting using the internet to complete some of their daily activities increased by 23% between the first two waves of the OWLS study, from 65.8 to 80.8%. This means that a significant proportion of people that were offline at the outbreak of the pandemic transitioned to Internet users in less than a year. However, a plateau was reached by the third wave of the study with only a 1.7% increase. It seems as if by that point, most people who were able to shift from offline to online had already completed the transition. Furthermore, they did not regress back to their offline status at the phaseout of the pandemic. Similar trends were found in the general UK population [32] wherein 1.5 million new Internet users emerged in 2021 compared to 2020 and 91% reported intending to continue with their new online activities post-pandemic. Among British people, 53% admitted they would not have coped through the pandemic without digital technology, underlying the urgency and necessity for digital engagement during the pandemic, which might also explain the relatively quick transition we observed in this study. However, other societal groups with traditionally low digital engagement, such as older adults, did not show a significant increase in use of the Internet over the course of the COVID-19 pandemic in England [33].

In the general population, 7% reported not using the Internet and were thus deemed as digitally excluded [34]. The stricter conceptualization of digital exclusion in this case compared to our study (i.e., people using the Internet or not as opposed to being able to meet their digital needs or not) does not allow for direct comparisons. However, 18% of our participants reported not using the Internet, suggesting that a digital divide might exist.

Perceived digital skills appeared to decrease since the beginning of the pandemic. More people in this study perceived their Internet knowledge as fair or worse than fair (54.4%) compared to our early-pandemic findings (45.7% - Spanakis, Heron, et al., [10]). Considering the increase in Internet use during the pandemic, the opposite pattern was expected. However, this finding might be explained by two factors: First, increased digital engagement might have revealed deficits in Internet use that people were not initially aware of, and second, the influx of new Internet users as the pandemic evolved might mean that our current sample included more people whose skills were at a beginner’s level.

Difficulty in concentrating was the most reported SMI-related symptom that obstructed the use of the Internet. The same has been found in earlier studies of people with schizophrenia [20]. A qualitative study also reported problems with concentration and information processing as a common struggle of people with SMI when using the Internet [35]. This finding supports calls to consider cognitive deficits and accommodate for them in designing online resources for people with SMI [36]. The second most reported symptom barrier was depressive episodes. This might be related to attention problems as well, which often occur during a depressive episode [37], or to motivation deficits in depression [38].

Our participants scored 26.0 in digital health literacy. A recent study exploring Digital Health Literacy in Greek and Finnish adults with SMI estimated eHEALS scores between 23.15 and 27.05 respectively and considered them to represent low to moderate literacy [20]. This implies that participants in our sample were moderately skilled to engage with health information online and applying this information to understand and self-manage their mental illness. Digital health literacy was higher among those with self-perceived advanced Internet knowledge, probably reflecting a greater level of digital engagement and confidence in using online resources. It was also found that people with bipolar disorder had greater digital health literacy compared to those with psychosis spectrum disorders. This is in line with previous findings [21] and adds to our previous findings that people with bipolar are more likely to frequently use the Internet [10] and less likely to lack foundation digital skills [17] compared to people with psychosis spectrum disorders. This suggests that digital exclusion may be more greatly experienced by people with psychosis spectrum disorders than people with bipolar affective disorder, potentially indicating that this subgroup may be in greater need of tailored digital engagement support.

### Implications and directions for future research

Our current findings demonstrate the need for training programs to help people with SMI improve their digital skills and digital health literacy (see for example the Digital Opportunities for Outcomes in Recovery Service – DOORS program [39, 40]). The results indicated that those participants with greater levels of self-rated internet knowledge had higher levels of digital health literacy, thus demonstrating the potential benefits of improving general digital skills for those people with SMI who are digitally excluded. Future studies should therefore focus on designing programs that consider the special needs of people with SMI and assessing the effectiveness of such programs in improving digital skills. Such studies should also explore what people with SMI want to use the Internet for and what would be the most preferable setting and methods for learning.

There are also some questions that were not answered in our study and may be the focus of future studies. To better understand who adapted to the digital demands of the Covid-19 pandemic, studies should explore the demographic, social, and health-related characteristics of those whose Internet use increased over the course of the pandemic. Differences in digital exclusion among SMI diagnoses should also be investigated. Qualitative studies are needed to explore the online experiences of people with SMI (e.g., what do they use the Internet for and how Internet use affects wellbeing), and to examine their needs and preferences for receiving in person and telephone communications as alternatives to digital access.

### Limitations

This study’s results should be interpreted with consideration to some limitations. For instance, we recruited participants only from England, not including other nations within the United Kingdom or internationally. In addition, when comparing participants to non-participants, people of White background were more likely to participate in the study, although there were no other sociodemographic differences. We were unable to compare participants to non-participants in terms of their current health status and thus we cannot rule out the possibility that the sample comprised of people that were less severely affected by their SMI conditions at the time of the study (i.e., the “healthy population effect”). These issues might limit the generalizability of the findings to international SMI samples or people currently undergoing a more severe phase of their illnesses. Indeed, it may be that non-participants were experiencing more severe mental ill health and had lower levels of digital skills and digital health literacy. If this were to be the case, we may be underestimating the extent of digital exclusion in this study. Moreover, knowledge about the Internet, improvement in Internet skills over the pandemic, and symptomatic barriers to Internet use were measured via self-report rather than objective observations (e.g., a skills evaluation). As such, our findings represent people’s self-perceptions and level of insight on these matters.

## Conclusion

Despite the use of the Internet increasing during the pandemic among people with SMI, sizeable proportions report moderate to low perceived digital skills as well as experiencing digital exclusion. The level of digital health literacy within the sample was also moderate, despite the long-term health conditions in this population. These findings underline the need for training and support among people with SMI to increase digital skills and further facilitate digital engagement as well as facilitating inclusion by offering non-digital engagement options to service users with SMI.

### Electronic supplementary material

Below is the link to the electronic supplementary material.

Additional File 1: Copy of the survey completed by participants in OWLS 3.

Additional File 2: Complete Case Analyses: Results of analysis including only participants with complete cases, without using data imputation techniques.

## Data Availability

The datasets generated and/or analysed during the current study are not publicly available due to ethical concerns. We have not obtained consent from participants to share their data other than for research purposes only. On that basis, data are available from the corresponding author on reasonable request.
