# Supplementary material for: Digital health literacy and digital engagement for people with severe mental ill health across the course of the COVID-19 pandemic in England
Source: BMC Med Inform Decis Mak. 2023 Sep 26;23:193. doi: 10.1186/s12911-023-02299-w (PMC10523616; doi:10.1186/s12911-023-02299-w)
Supplement: Supplementary file 1 — Additional File 1: Copy of the survey completed by participants in OWLS 3. [file 12911_2023_2299_MOESM1_ESM.pdf]

UNIVERSITY of York

OWLS³

# Optimising Wellbeing during Self-isolation

Thank you for agreeing to take part in this study.

# 1. Your Health and Wellbeing

## In this section we would like to ask you about your physical health, mental health and wellbeing.

**1.1 We would like to ask you four questions about how you are feeling.**

There are no right or wrong answers. For each of these questions we would like you to give an answer on a scale of 0 to 10, where 0 is 'not at all' and 10 is 'completely'.

| | 0 | 1 | 2 | 3 | 4 | 5 | 6 | 7 | 8 | 9 | 10 |
|---|---|---|---|---|---|---|---|---|---|---|---|
| 1.1a Overall, how satisfied are you with your life nowadays? | | | | | | | | | | | |
| 1.1b Overall, to what extent do you feel that the things you do in your life are worthwhile? | | | | | | | | | | | |
| 1.1c Overall, how happy did you feel yesterday? | | | | | | | | | | | |

**1.2 Overall, how anxious did you feel yesterday?**

Please answer on a scale of 0 to 10, where 0 is 'not at all anxious' and 10 is 'completely anxious'.

| | 0 | 1 | 2 | 3 | 4 | 5 | 6 | 7 | 8 | 9 | 10 |
|---|---|---|---|---|---|---|---|---|---|---|---|
| Overall, how anxious did you feel yesterday? | | | | | | | | | | | |

**1.3 Over the last 2 weeks, how often have you been bothered by the following problems?**

| | Not at all | Several days | More than half the days | Nearly every day |
|---|---|---|---|---|
| 1.3a Little interest or pleasure in doing things | | | | |
| 1.3b Feeling down, depressed or hopeless | | | | |

**1.4 Over the last 2 weeks, how often have you been bothered by the following problems?**

| | Not at all | Several days | More than half the days | Nearly every day |
|---|---|---|---|---|
| 1.4a Feeling nervous, anxious or on edge | | | | |
| 1.4b Not being able to stop or control worrying | | | | |

**1.5 Do you have any physical health conditions?**

☐ Yes ☐ No

**Heart condition (such as heart disease, heart failure)**

☐ Yes ☐ No

**Lung condition (such as COPD, asthma, lung cancer)**

☐ Yes ☐ No

**Diabetes**

☐ Yes ☐ No

**Digestive condition (such as conditions affecting the stomach and bowel)**

☐ Yes ☐ No

**Heart condition (such as heart disease, heart failure)**

☐ Yes ☐ No

**High blood pressure**

☐ Yes ☐ No

**Neurological condition (such as epilepsy, Parkinson's)**

☐ Yes ☐ No

**Chronic pain**

☐ Yes ☐ No

**Kidney disease**

☐ Yes ☐ No

**Other/s please state below:**

## 2. Service use

**2.1 Do you think that you will need more help or support from NHS mental health services in the next year?**

☐ Yes ☐ No ☐ Maybe

**2.2 Do you think that you will need more help or support from charities or other non-NHS organisations for your mental health in the next year?**

☐ Yes ☐ No ☐ Maybe

**2.3 If you responded yes or maybe to questions 2.1 or 2.2 please briefly describe below what type of help or support you think you will need.**

| |
|---|
| |

## 3. Everyday habits

In this section we would like to ask about daily habits and routines. Everyone has healthy habits and unhealthy habits and there are no right or wrong answers.

**3.1 How important is it that you maintain a healthy lifestyle?**

☐ A top priority
☐ Moderately important
☐ I don't worry about it

**3.2.1 In general, how often do you take part in any sport or physical activity?**

☐ Every day ☐ Every other day ☐ At least once a week
☐ More than once a month but less than once a week
☐ Less than once a month ☐ Never

**3.2.2 Would you like to take more exercise?**

☐ Yes ☐ No ☐ Don't know

**3.3.1 In general, how many portions of fruit and vegetables do you eat per day?**

☐ I don't eat fruit or vegetables

☐ One ☐ Two ☐ Three ☐ Four ☐ Five or more

**3.3.2 Would you like to change you diet?**

☐ Yes ☐ No ☐ Don't know

**3.3.3 Would you like to lose or gain some weight?**

☐ Yes, I would like to **gain** weight ☐ Yes, I would like to **lose** weight

☐ No ☐ Don't know

**3.4a Do you smoke tobacco?**

☐ Yes ☐ No

Please answer 3.4b to 3.4e ONLY if you smoke (not counting e-cigarettes). Otherwise go straight to 3.5.

**3.4b How many cigarettes per day do you usually smoke? (Write a number below)**

............ per day

**3.4c How many cigarettes per day do you usually smoke (Please choose an option)**

☐ 10 or less ☐ 11 to 20 ☐ 21 to 30 ☐ 31 or more

**3.4d How soon after you wake up do you smoke your first cigarette?**

☐ within 5 minutes ☐ 6-30 minutes ☐ More than 30 minutes

**3.4e Would you like to cut down or quit smoking?**

☐ Yes ☐ No ☐ Don't know

**3.5 Do you use an e-cigarette or vape?**

☐ Yes ☐ No

**3.6 In the LAST MONTH, have you been able to maintain a daily routine in your life?**

☐ more than usual ☐ about the same ☐ less than usual

**3.7 How often did you have a drink containing alcohol in the past year?**

☐ Never ☐ Monthly or less ☐ Two to four times a month

☐ Two to three times per week ☐ Four or more times a week

**3.8 How many drinks containing alcohol did you have on a typical day when you were drinking in the last year?**

☐ 1 or 2 ☐ 3 or 4 ☐ 5 or 6 ☐ 7 to 9 ☐ 10 or more

**3.9 How often did you have six or more drinks in the past year?**

☐ Never ☐ Less than monthly ☐ Monthly ☐ Weekly ☐ Daily or almost daily

# 4. Social support

## In this section we would like to ask you about the support you receive from people around you.

**4.1 Thinking of the LAST TWO WEEKS, please choose an option for each of the questions below.**

| | hardly ever | some of the time | often |
|---|---|---|---|
| 4.1a How often do you feel that you lack companionship? | | | |
| 4.1b How often do you feel left out? | | | |
| 4.1c How often do you feel isolated from others? | | | |
| 4.1c How often do you feel lonely? | | | |

**4.2 Thinking about yourself in general, please chose an option for each question below*.**

| | Strongly disagree | Disagree | Neutral | Agree | Strongly agree |
|---|---|---|---|---|---|
| 4.2a I tend to bounce back quickly after hard times | | | | | |
| 4.2b I have a hard time making it through stressful events. | | | | | |
| 4.2c It does not take me long to recover from a stressful event. | | | | | |
| 4.2d It is hard for me to snap back when something bad happens. | | | | | |
| 4.2e I usually come through difficult times with little trouble. | | | | | |
| 4.2f I tend to take a long time to get over set-backs in my life. | | | | | |

*Smith, B.W., Dalen, J., Wiggins, K., Tooley, E., Christopher, P. and Bernard, J. (2008). The Brief Resilience Scale: Assessing the Ability to Bounce Back. International Journal of Behavioral Medicine,15, 194-200.

# 5. Use of the internet and digital devices

## Here we ask if/how you have used the internet and digital devices.

**5.1 In the last 12 MONTHS, have you used the internet to do some of your daily activities?** (e.g. for video-calling, buying groceries, paying bills, finding information etc.)

☐ Yes, a lot ☐ Yes, a little ☐ No

**5.2 Please state your level of agreement with the following sentence:** ***I have been using the internet mainly for social media*** (e.g. Facebook, Instagram, Twitter, etc.) or messaging (e.g. Messenger, Whattsapp, etc)

☐ Yes ☐ No ☐ Not applicable (I am not going online)

**5.3 How would you rate your knowledge about the Internet?**

☐ Outstanding ☐ Good ☐ Fair

☐ Poor ☐ Bad ☐ Don’t know/can’t say

## If your answer to 5.3 was ‘Fair’ or lower please complete the following:

**5.4 Indicate your level of agreement with the following statements**

| | Strongly disagree | Disagree | Neutral | Agree | Strongly agree |
|---|---|---|---|---|---|
| I know that if I worked hard to learn about computers/Internet, I could do well’ | | | | | |
| Computers/Internet are too complicated for me to understand | | | | | |
| I think I am the kind of person who would learn to use a computer/Internet well | | | | | |
| I think I am capable of learning to use a computer/Internet | | | | | |
| Given a little time and training, I know I could learn to use a computer/Internet | | | | | |
| Learning about computers/Internet is a worthwhile and necessary subject | | | | | |
| Reading or hearing about computers/ Internet would be (is) boring | | | | | |
| I don’t care to know more about computers/ Internet | | | | | |
| Computers/Internet would be (are) fun to use | | | | | |
| Learning about computers/ Internet is a waste of time | | | | | |

**5.5 If you were to learn new digital skills, which of the following would be important to you?**

| | Yes | No |
|---|---|---|
| It needs to be organised or recognised by my place of work | | |
| It needs to be 'live' so I can ask questions | | |
| An industry-recognised qualification at the end of the course is important to me | | |
| It needs to be led by a digital expert or trainer | | |
| It needs to be free for me to take part | | |
| I prefer to learn the content by reading user guides in my own time | | |
| I would prefer face-to-face as and when it becomes available again | | |
| I would prefer to learn from friends, family or work colleagues | | |
| I prefer recorded tutorials, so I can play back content if needed | | |
| I need to learn by having a go myself | | |

**5.6 Do you think your digital skills have improved as a result of the outbreak of the Coronavirus crisis?**

☐ Yes ☐ No, although I do feel that they need improving
☐ No, but I do not feel they need improving
☐ Don't know / Prefer not to say

**5.7 Was there ever a time that you could not do something that you needed/wanted because you were not able to use the Internet or digital technologies?**

☐ Yes ☐ No

**5.7.1 If 'Yes', please give us an example**

……………………………………………………………………..

**5.8 Have any of the following ever obstructed your use of the Internet?**

| | Almost never | A few times | Many times |
|---|---|---|---|
| Experiencing visual hallucinations | | | |
| Hearing voices | | | |
| Finding it difficult to concentrate for long periods of time | | | |
| Experiencing paranoid ideas | | | |
| Experiencing an episode of depression | | | |
| Experiencing an episode of mania | | | |
| Pain in limbs | | | |
| Unsteady hands | | | |
| Difficulty sitting for long period of time | | | |
| Eyes that tire easily | | | |
| Other | | | |

**5.8.1 If 'Other', please describe**

…………………………………………………………………………..

**5.9. Please indicate your level of agreement with the following statements.**

| | Strongly disagree | Disagree | Neutral | Agree | Strongly agree |
|---|---|---|---|---|---|
| I know what health resources are available on the Internet | | | | | |
| I know where to find helpful health resources on the Internet | | | | | |
| I know how to find helpful health resources on the Internet | | | | | |
| I know how to use the Internet to answer my questions about health | | | | | |
| I know how to use the health information I find on the Internet to help me | | | | | |
| I have the skills I need to evaluate the health resources I find on the Internet | | | | | |
| I can tell high quality health resources from low quality health resources on the Internet | | | | | |
| I feel confident in using information from the Internet to make health decisions | | | | | |

## 6. Any other comments

In this section you can tell us about anything else you think is important for you.

**6.1 Is there anything else you would like to add?**

☐ No, no further comments

☐ Yes (please type your comments below)

## Thank you for taking part

Your participation was valuable to understand the impact of the pandemic restrictions on people's health and wellbeing.

By returning this survey you are indicating that you agree to your answers to this questionnaire being recorded and consent to taking part in this study and being a member of the OWLS Cohort.

You also consent for the anonymised data you have provided in your responses to be shared with other researchers for research purposes only.

**For internal use only**

If you are a researcher and you have any comments to add please enter them here.
