# Supplementary material for: Digital health literacy and digital engagement for people with severe mental ill health across the course of the COVID-19 pandemic in England
Source: BMC Med Inform Decis Mak. 2023 Sep 26;23:193. doi: 10.1186/s12911-023-02299-w (PMC10523616; doi:10.1186/s12911-023-02299-w)
Supplement: Supplementary file 2 — Additional File 2: Complete Case Analyses: Results of analysis including only participants with complete cases, without using data imputation techniques. [file 12911_2023_2299_MOESM2_ESM.docx]

**Additional File 2 - Complete Case Analyses: Results of analysis including only participants with complete cases, without using data imputation techniques.**

**Internet Use**

A total of *n*=141 participants (84.9% after excluding *n*=11 with missing data) reported using the Internet to do some of their daily activities. When analysing the *n*=146 participants who had reported whether they used the Internet daily or not in each of the three OWLS surveys, a Cochran’s Q test identified a significant difference between the three time points in terms of proportions of Internet use (*Q*=35.2, *df*=2, *p*<.001). Specifically, post-hoc pairwise McNemar tests identified that participants reported using the Internet significantly less (both *p*<.001) during OWLS 1 (65.8%) than during OWLS 2 (80.8%) or OWLS 3 (84.9%). No difference was found between OWLS 2 and OWLS 3 (*p*=0.134).

**Digital Health Literacy**

Before conducting the multiple linear regression to investigate factors associated with Digital Health Literacy, *n*=35 participants were excluded. This is due to *n*=32 participants not having complete information, and only *n*=3 participants being transgender, thus resulting in there being too few participants of this gender to enable appropriate analysis. Consequently, data from a total of *n*=142 participants were available, and Table S1 summarises the results from the conducted linear regression. It was found that having outstanding or good self-reported knowledge of the Internet and having a diagnosis of bipolar disorder (compared to psychosis spectrum disorder) were significantly associated with having higher levels of Digital Health Literacy. In contrast to the multiple linear regression model that used imputed data, being female was not found to be significantly associated with Digital Health Literacy, while age was found to be significantly associated.


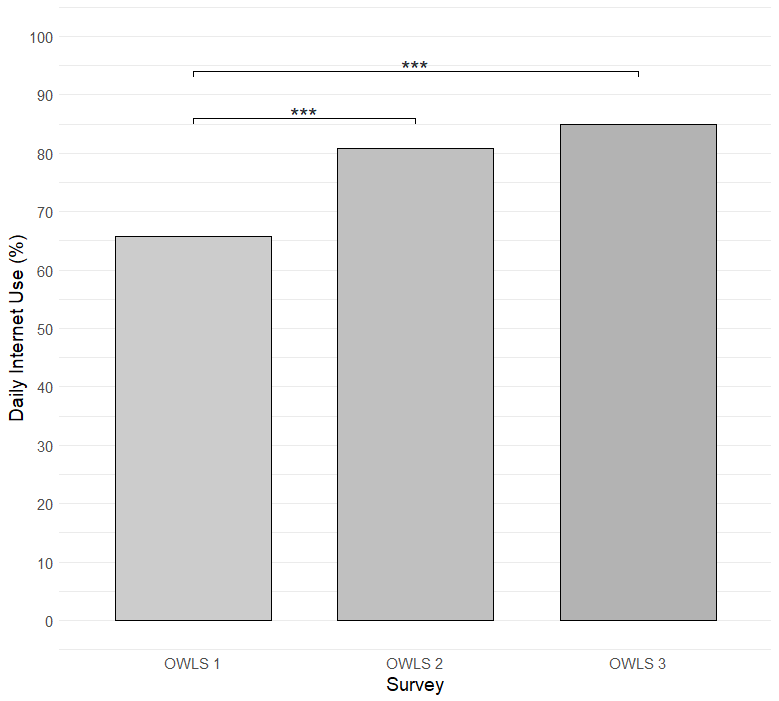


**Figure S1.**

*Proportions of participants in each OWLS survey who report using the Internet to do some of their daily activities at least some of the time (n = 146; complete data)*

*** indicates statistically significant difference between two proportions (p < 0.001).

| **Table S1.**  ***Factors associated with Digital Health Literacy (n=142).*** | | | | | |
| --- | --- | --- | --- | --- | --- |
|  | Estimate | SE | 95% CI | | p |
|  |  |  | LL | UL |  |
| Intercept | 24.10 | 3.48 | 17.21 | 30.99 | <0.001* |
| Age | -0.12 | 0.06 | -0.23 | <0.01 | 0.043* |
| Gender - Female | 2.76 | 1.49 | -0.19 | 5.70 | 0.066 |
| Ethnicity – Other than White | -1.21 | 2.31 | -5.79 | 3.37 | 0.603 |
| Index of Multiple Deprivation | 0.48 | 0.53 | -0.58 | 1.53 | 0.371 |
| Diagnosis - Bipolar | 4.33 | 1.58 | 1.20 | 7.46 | 0.007* |
| Diagnosis – Other SMI | 1.09 | 2.58 | -4.02 | 6.20 | 0.673 |
| Physical Health Problem – Having One | -0.04 | 1.61 | -3.22 | 3.15 | 0.983 |
| Cumulative Index of Health | 0.83 | 0.97 | -1.10 | 2.75 | 0.397 |
| Internet Knowledge – Outstanding/Good | 5.60 | 1.64 | 2.36 | 8.84 | <0.001* |
| *Statistically significant when tested against an alpha value of 0.05. | | | | | |
